# Dynamic treatment effect (DTE) curves reveal the mode of action for standard and experimental cancer therapies

**DOI:** 10.18632/oncotarget.4141

**Published:** 2015-05-15

**Authors:** Kingshuk Roy Choudhury, Stephen T. Keir, Kathleen A. Ashcraft, Mary-Keara Boss, Mark W. Dewhirst

**Affiliations:** ^1^ Department of Biostatistics and Bioinformatics, Duke University Medical Center, NC, USA; ^2^ Preston Robert Tisch Brain Tumor Research Center, Duke University Medical Center, NC, USA; ^3^ Department of Radiation Oncology, Duke University Medical Center, NC, USA; ^4^ North Carolina State College of Veterinary Medicine, Department of Molecular Biomedical Sciences, NC, USA

**Keywords:** tumor growth delay, tumor growth modelling, inhibition rate, regrowth following radiotherapy, additive effects in combination therapy

## Abstract

We present a method for estimating the empirical dynamic treatment effect (DTE) curves from tumor growth delay (TGD) studies. This improves on current common methods of TGD analysis, such as T/C ratio and doubling times, by providing a more detailed treatment effect and overcomes their lack of reproducibility. The methodology doesn't presuppose any prior form for the treatment effect dynamics and is shown to give consistent estimates with missing data. The method is illustrated by application to real data from TGD studies involving three types of therapy. Firstly, we demonstrate that radiotherapy induces a sharp peak in inhibition in a FaDu model. The height, duration and timing of the peak increase linearly with radiation dose. Second, we demonstrate that a combination of temozolomide and an experimental therapy in a glioma PDX model yields an effect, similar to an additive version of the DTE curves for the mono-therapies, except that there is a 30 day delay in peak inhibition. In the third study, we consider the DTE of anti-angiogenic therapy in glioma. We show that resulting DTE curves are flat. We discuss how features of the DTE curves should be interpreted and potentially used to improve therapy.

## INTRODUCTION

A tumor growth delay (TGD) experiment is often the last step in preclinical cancer drug development. When a therapy that has shown efficacy in *in vitro* studies fails to repeat effects in a TGD study, we would like to know why. However, common methods for reporting results from TGD studies do not provide any information regarding mechanisms failure, because they merely provide an overall measure of efficacy of a therapy. Typical results do not provide any information as to what methods could be modified to improve efficacy. Here, we describe a new analysis method for TGD studies that can be used as an investigative tool, rather than just for screening.

**Figure 1:**
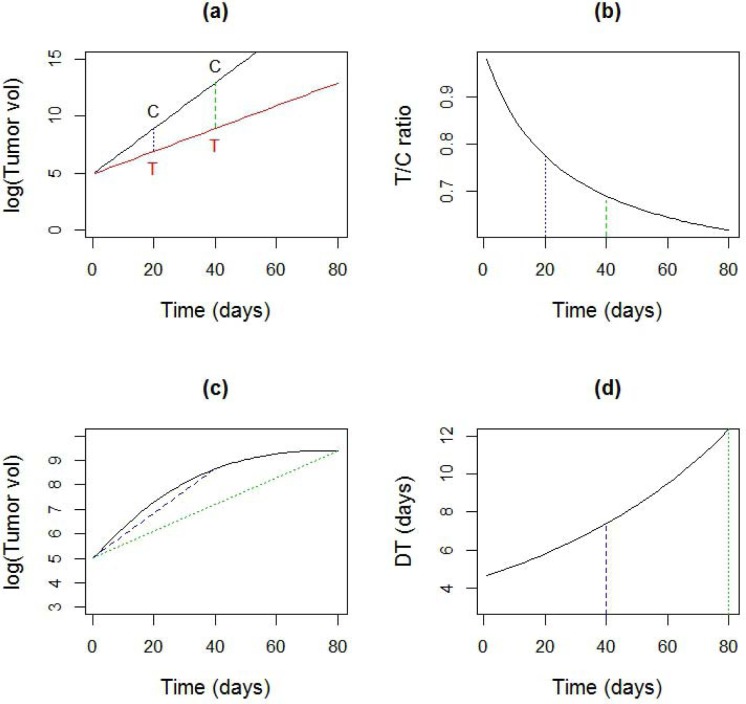
Sensitivity of common summary measures to time **a.** Log-linear tumor growth curves for data in control (C) and treated (T) group with a growth rate of 20%/day and 10%/day respectively **b.** The time dependence of the T/C ratio for curves in **a. c.** A non-linear tumor growth curve **d.** Time dependence of doubling time (DT), calculated using two observations from the curve in **c.**, using the formula DT = log(2)/(log(*V*(*t*)) − log(*V*(0)).

Results from TGD studies often lack reproducibility [1]. One reason for lack of reproducibility is the use of single number summaries to capture the treatment effect. For instance, the value of the T/C ratio, a widely used measure, is strongly dependent on the time at which the ratio is calculated (Figure 1(a)-1(b)). The comparison time depends on when tumor burdens from ‘most’ animals in the group are observable, which in turn, are driven by IACUC regulations. Due to inter animal variation in growth, this time can be subject to considerable randomness, causing lack of reproducibility. Another commonly used measure, tumor doubling time, is usually calculated using tumor volumes at two time points [2]. While doubling time does give consistent results under log-linear growth, which works for control tumors [3], consistency is lost under non-linear growth (Figure 1(c)-1(d)), which is typically seen in treatment arms. The time dependence of these single number summaries highlights the need for a time varying (dynamic) estimate of the treatment effect.

Where feasible, pharmacokinetic-pharmacodynamic (PK-PD) modelling can provide a ‘mechanistic’ understanding of drug effects on tumor growth [4]. However, such modelling is often based on assumptions about key rate parameters and/or requires measurement of a validated target inside the tumor [5], which can be quite expensive or difficult to obtain. This is particularly true when novel drugs, whose mechanism of action *in vivo* are as yet unknown, are considered. Other problematic situations include radiotherapy, where PK measurements aren't meaningful or combination therapy, where again the operational target for PD isn't clear. An alternative approach to analysis of TGD studies is by fitting curves to growth profiles. Various forms of curves, such as linear in dose [6], linear exponential mixtures [7] and recently, multi-phase growth models have been proposed [8, 9]. While these models may fit the data quite well, one problem many of these models share is that the coefficients have limited biological interpretation [10]. Interpretability is key to understanding why a therapy does or does not work and how it might be improved. Another limitation of model based analysis is that it typically assumes a particular type of treatment effect. With novel therapies and combinations, we will see that the form of the treatment effect can be hard to predict. The holy grail in TGD modelling is therefore to develop a method that i) fits the data well for a wide variety of cancers and therapies without detailed knowledge of their mechanism of action and ii) provide results that are biologically interpretable and actionable.

**Figure 2:**
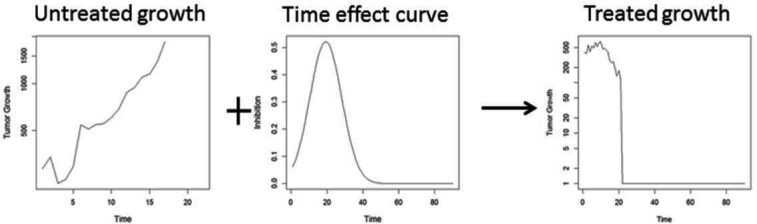
Superposition of two processes governing tumor growth dynamics: uncontrolled growth + inhibitory treatment effect lead to observed tumor growth delay (TGD) curves The goal is to estimate the dynamic treatment effect curve from observed TGD curves in treated and untreated animals.

Tumor growth under treatment can be thought of as the superposition of two processes: a) a growth process *G*(*t*), that occurs when tumors are untreated and b) an inhibition process or *R*(*t*), which is the treatment effect (Figure 2, equation (1.1)). The key idea of this paper is to estimate and interpret these two processes to gain insight into the treatment effect. The inhibition process can be estimated in this general form using methods for ‘nonparametric’ or ‘functional’ regression [11]. This allows us to examine the empirical dynamics of the treatment effect. As we shall see in the examples, the dynamics of the treatment effect reveal a lot more about the mode of action of a therapy than can be captured by any single summary measure.

(1.1) V(t)=V(0)exp(G(t)−R(t))

## RESULTS

### Simulation experiment

The accuracy of the estimated treatment effects was assessed in a simulation experiment. TGD data were generated with *n* = 10 animals per treatment group, observed every third day over a period of 30 days. Data was generated from the general growth model (1.2). Each animal was assigned a random initial tumor volume *V*_i_(*0*) by sampling from a log normal distribution with mean 100 mm^3^ and SD 20 mm^3^. Measurement error ε_i_ followed a Gaussian distribution with zero mean and SD 20 mm^3^. Data were censored if the observed tumor volumes *V_i_*(*t*) fell below 20 mm^3^ or exceeded 2000 mm^3^. The control growth rate λ_i_ was sampled from a Gaussian distribution with mean 14%/day and SD 2%/day. The ‘true’ treatment effect curve was of the form $R(t)=a\exp(-0.5{\left(t-\mu \right)}^{2}/\sigma^{2}){\left(2\pi \right)}^{-1}\sigma$, with peak location μ sampled from a Gaussian distribution with mean 20 days and SD 2 days, duration σ sampled from a Gaussian distribution with mean 10 days and SD 1 day. Two different values of scale were used i) *a* = 5, which generated some shrinkage followed by regrowth (Figure 4a) ii) *a* = 15, which led the tumor to become unobservable followed by occasional regrowth (in other cases the tumor vanished) (Figure 4c). The values used for the simulation produce growth profiles typical for real TGD studies.

**Figure 3:**
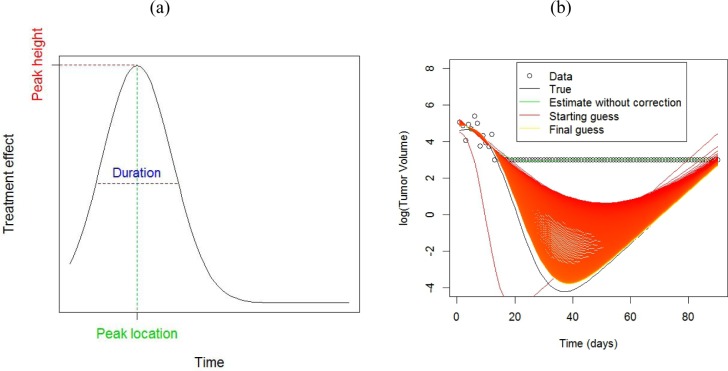
**a.** Features of a treatment effect curve **b.** Example of the use of the EM algorithm for estimation with censored data, using a scaled Gaussian density as model for the treatment effect curve. The black line shows a ‘true’ growth curve, whereby the tumor shrinks considerably before growing again. Tumor volumes below 20 mm^3^ were not palpable and these observations are set to 20 mm^3^. The green line shows the estimated growth curve by fitting a smoothing spline using the recorded data. The EM algorithm (red line) imputes data values for observations below 20 mm^3^. Successive iterates (colored on a graduated scale from red-orange-yellow) from the EM algorithm improve upon the estimated growth curve to come quite close to the true value by the 1000^th^ iteration.

**Figure 4:**
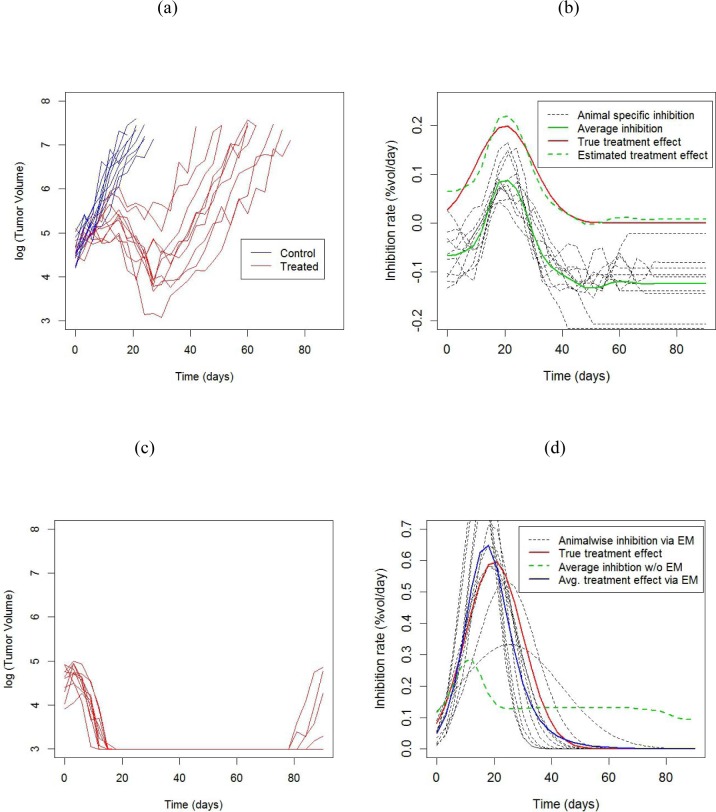
Results from simulation experiment **a.** Tumor growth profiles from a two armed study. Each line represents an animal. **b.** Animalwise and average inhibition rates estimated from the data in **a.**, obtained using the non-parametric (model free) method. The treatment effect curve is obtained by subtracting the growth rate in the control group from the average inhibition curve. **c.** Tumor growth profiles from a treatment group showing large tumor shrinkage with occasional regrowth. The control arm is the same as in **a.**. **d.** Animalwise and average inhibition rates estimated from the data in c) obtained using the Gaussian scaled density model, obtained via the EM algorithm to account for censored data. The average treatment effect obtained from non-parametric estimation, which doesn't take censoring into account, is also shown for comparison.

The average growth rate in the control group was estimated by fitting a linear mixed model to the log volumes [7] as $\bar{\lambda }$ = 0.13, with an SD of 0.02 across animals. For the moderate shrinkage data in Figure 4(a), we used the non-parametric estimate given in equation (1.5). Although the peak is well estimated, there appears to be some bias beyond 50 days (Figure 4(b)), because the growth curves are all censored by that point (Figure 4(a)). For the high shrinkage data in Figure 4(c), using the non-parametric estimate gives a highly biased estimate of the treatment effect, due to the heavy censoring present in this dataset. By contrast, the EM algorithm gives an approximately unbiased estimate of the treatment effect (Figure 4(d)).

### Radiation therapy study

**Figure 5:**
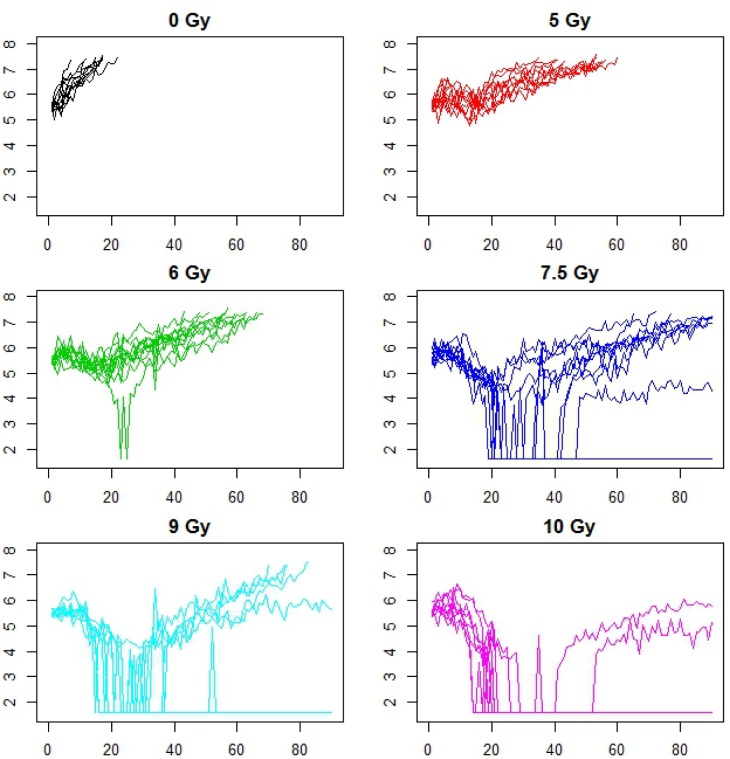
Animalwise tumor growth profiles for a dose escalation study using FaDu xenografts receiving varying levels of fractionated radiation therapy, between 0-10 Gy/day for days 1-5 There were between 8 - 11 animals per treatment group. The x axis is time in days, while the y axis is log tumor volume (mm^3^) for all plots. Axes labels are omitted for better resolution of curves.

**Figure 6:**
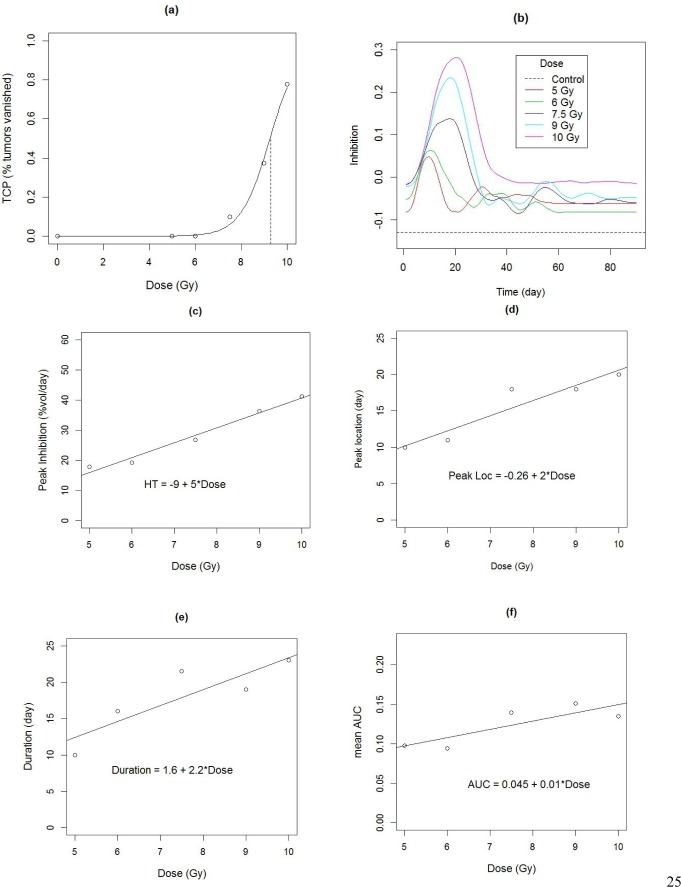
**a.** Dose response curve fitted to the empirical tumor control probability. The dashed vertical line indicates the TCD_50_ dose at 9.3 Gy. **b.** Estimated dynamic treatment effect curves for each arm of radiation therapy study, obtained from data in Figure 5. **c.- f.** Characteristics of treatment effect curves, as a function of dose. Lines shows fitted trend. **c.** Peak heights **c.** Peak location **d.** Peak duration **f.** Mean Area under the curve.

Radiation therapy is a commonly used treatment for head and neck cancer. Here we consider data from a study involving FaDu xenografts transplanted into nude mice. This xenograft was derived from a patient with head and neck cancer [23]. When tumor volumes reached 200-300 mm^3^, mice were randomized into 6 radiation treatment groups, with between *n* = 8-11 animals/group. Radiation fractions at doses ranging from 0 Gy (control) to 10 Gy were administered on 5 consecutive days. Tumors were measured daily with calipers, and volumes were calculated using the formula *V*=(*A*^2^*x*B*x*π)/6, where *A* is the shortest diameter and *B* is the longest diameter. Mice were sacrificed when their tumor reached 1500 mm^3^ (Figure 5). The standard method for analyzing dose escalation studies is a dose response curve, whereby the tumor control probability is modelled as a function of dose [24]. From the fitted curve, we find that the TCD_50_ dose (the radiation dose that controls 50% of the tumors) is 9.3 Gy with a standard error of 0.03 Gy and the steepness of the curve is 3.62 (SE = 0.33) (Figure 6 (a)). What additional insight can be gained from dynamic treatment effect curves?

The rate of growth in the control group was 13%/day with an SD of 2%/day across animals. Animalwise dynamic treatment effect (DTE) curves were obtained using the non-parametric or model based methods, depending on the extent of missing data (Figure 5), and then averaged across animals. Average DTE curves for all treatments exhibit a single peak, with peak size appearing to increase with dose (Figure 6(b)). We also observe that although the treatment effect is substantially diminished by 30 days, growth is still inhibited beyond this point, i.e. the regrowth rate is slower than the rate of control growth, as previously demonstrated in [25]. Next we characterized the DTE curves in terms of their salient features, namely peak height, peak location, duration (Figure 3a) as well as mean AUC, which represents the area under the curve per day of observation. We found a significant increasing trend in the peak height with dose at 5%/day/Gy (p-value = 0.001), from 15%/day at 5 Gy to 40%/day at 10 Gy (Figure 6(c)). Similarly there was an increasing trend in peak location, with a delay of about 2days/Gy (p-value = 0.02) from 10 days at 5 Gy to 20 days at 10 Gy (Figure 6(d)). Duration ranged from 10 days at 5 Gy to 21 days at 10 Gy. The trend in duration was marginally significant at 2.2 days/Gy (p-value = 0.05), although the duration at 5 Gy appears to be lower (2.9 days) than the rest (Figure 6(e)). There was no significant trend in mean AUC (mean = 12.3%/day, SD = 3%/day, p-value for trend = 0.08), although the mean AUC at 5 and 6 Gy appears to be lower than the rest (Figure 6(f)).

### Combination therapy

Combination therapy is quite common in cancer treatment, particularly when no single therapy is successful for a particular type of cancer. Here we consider a four armed experiment (*n* = 9 animals per group) on a patient derived high grade glioma xenograft. Apart from a control arm, the study had an arm treated with temozolomide, which is a standard therapy for glioma, on the first 5days at 25mg/kg IP, a third arm treated with an experimental drug which inhibits the BMI1 gene, which is a target to prevent tumor self-renewal [26], at maximum tolerated dose (MTD) PO twice weekly x 5, an and a fourth arm treated with combination of the two agents given at same doses and schedules.

**Figure 7:**
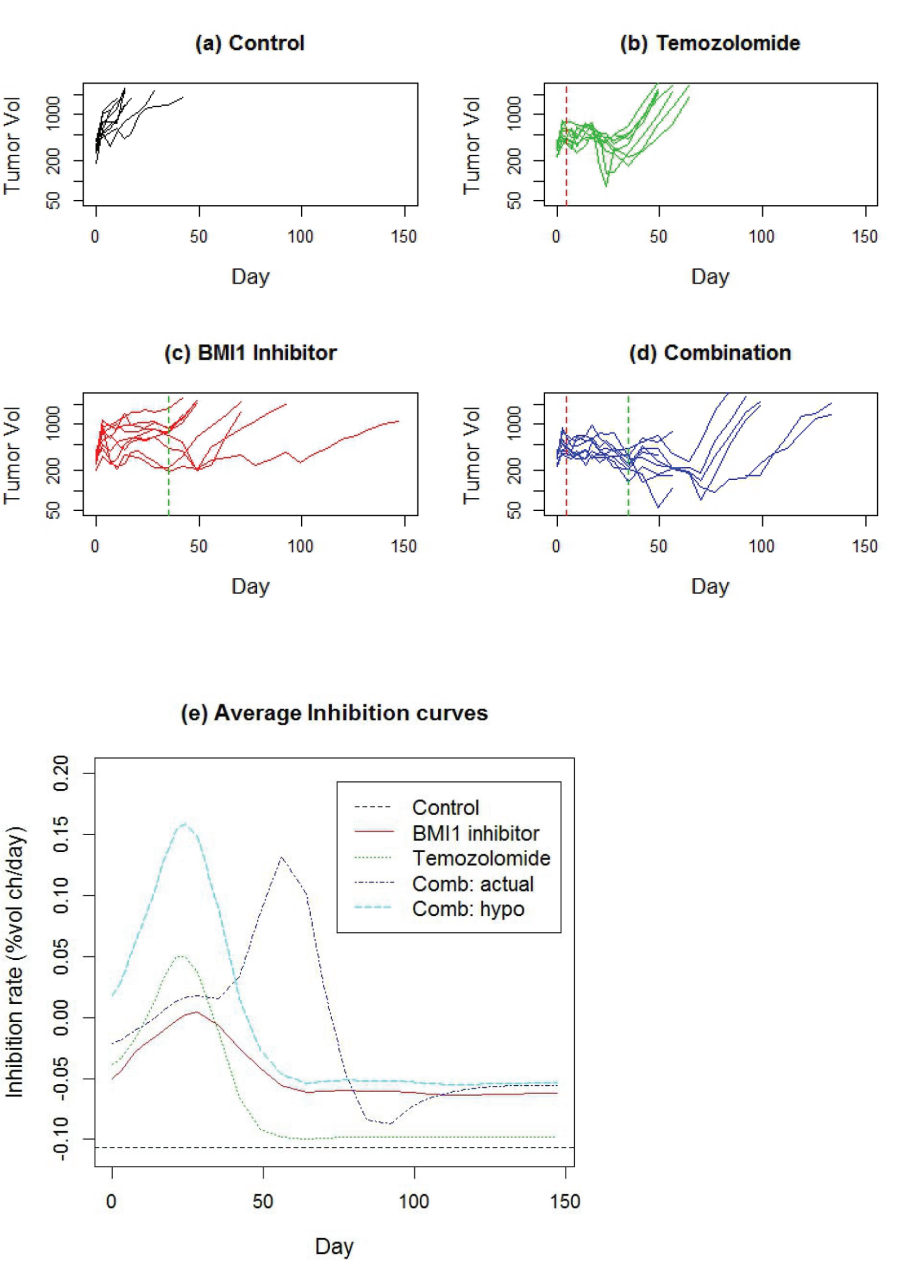
**a.-d.** Tumor growth profiles for a 4 arm study of patient derived glioma xenograft involving monotherapies of temozolomide and an experimental BMI1 as well as the combination of the two. The dashed vertical lines denote end of treatment. **e.** Estimated DTE curves for each treatment arm, as well as a hypothetical curve BMI1 inhibitor + Temo, representing the addition of the two mono-therapy DTEs.

The average rate of growth in the control group was 10.7%/day, with an SD of 4%/day across animals. Temozolomide inhibition peaked at day 28 (Table 1), but after day 50, tumors regrew at an average rate almost as fast as the control rate (Figure 7 (e)). The BMI1 inhibitor had lower peak inhibition than temozolomide, but the duration of BMI1 inhibitor (53 days) is substantially greater than that for temozolomide (32 days). For the combination therapy, if the effect of the treatments were ‘additive’, we would expect to see an inhibitory effect which is the sum of the DTE curves for the two mono-therapies, i.e. the sum of the red and green curves, shown in Figure 7(e) as the light blue curve. The actual effect of the combination therapy (dark blue curve) is similar to the hypothesized additive effect in terms of peak inhibition, duration and mean AUC (Table 1), however the location of the peak (56 days) is substantially delayed from what was hypothesized (24 days).

**Table 1:** Features of DTE curves for combination therapy study

Therapy	Peak inhibition (% vol/day)	Peak location (day)	Duration	AUC
Temozolomide	0.16	28	32	0.051
BMI Inhibitor	0.11	24	53	0.064
‘BMI Inhibitor + Temo’	0.27	24	38	0.115
Combination	0.24	56	49	0.095

### Anti-angiogenic therapy

**Figure 8:**
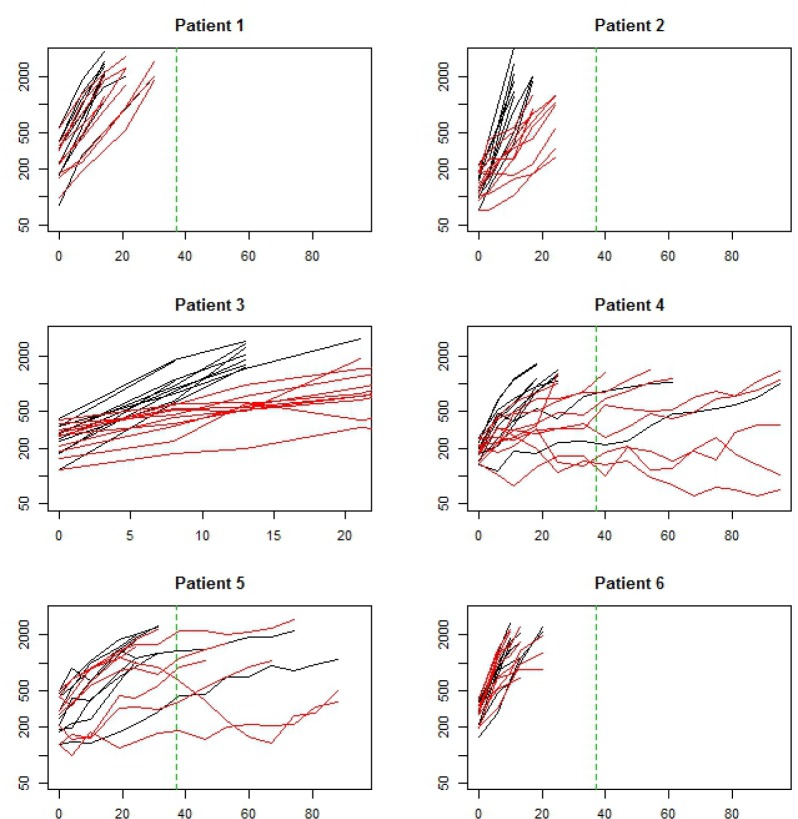
Data from a set of 6 two armed trials Black arm is control (no treatment). Red arm is Bevacizumab mono therapy. Each study used xenografts derived from a different patient with glioma. The x-axis is time (days) and the y-axis is log tumor volume. The vertical green dashed line represents end of therapy.

Anti-angiogenic therapies act by restricting blood supply to the tumor, thus reducing tumor growth. Bevacizumab is an anti angiogenic agent FDA approved for treatment of recurrent gliobastoma. Here we consider data from 6 studies, each with two arms and *n* = 10 animals/arm, one control and the other treated with 5mg/kg of Bevacizumab twice weekly x 5 weeks (Figure 8). Tumors in each study were derived from tumor tissue from a different patient with glioma.

**Figure 9:**
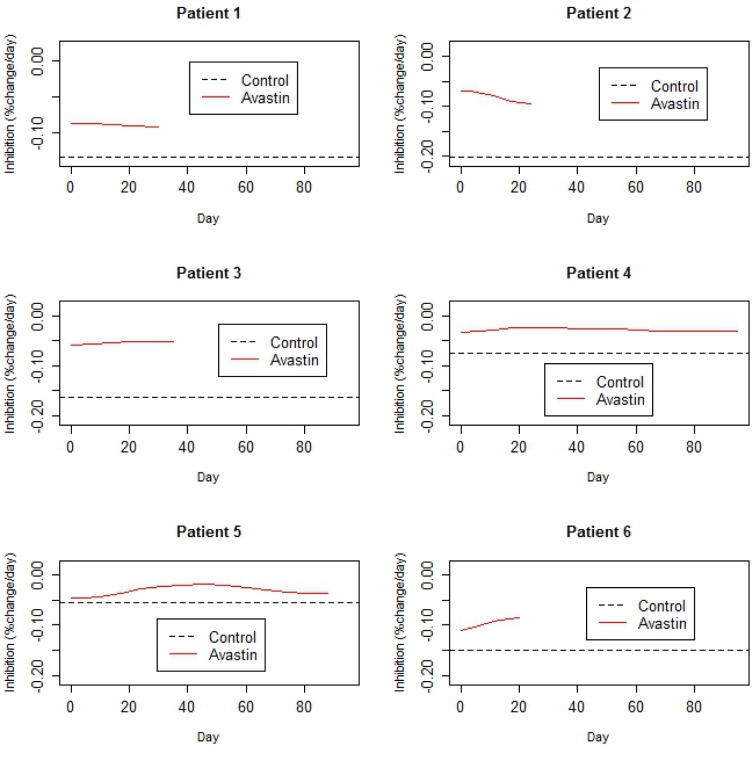
Estimated rates of control growth and inhibition due to Bevacizumab treatment, derived from the data in Figure 8

Growth rates in the control arm ranged between 5.4 %/day to 20.1%/day (Table 2). The estimated treatment effect curves were approximately flat for all 6 studies, indicating a relatively constant rate of inhibition throughout the study (Figure 9). Peak growth rates in the treated arm were negative across studies, implying that on average no tumor shrinkage took place at any point in the study. As might be expected, tumors in studies exhibiting lower growth rates lasted longer (Figure 8).

**Table 2:** Summary of growth and inhibition rates in Bevacizumab study

PDX ID	Control growth rate (%/day)	SD in growth rate across animals (%/day)	Inhibition effect of Avastin (%/day)
1	13.4	1.9	3.8
2	20.1	3.8	12.8
3	16.3	2.6	11.1
4	7.5	3.0	4.9
5	5.4	2.2	2.5
6	15.0	4.1	2.9
Mean	13.0	2.9	6.6
SD	5.5		4.5

## DISCUSSION

We have demonstrated that the treatment effect for some common types of cancer therapy show a strong non-linear time dependence, which we can estimate via the methodology proposed in this paper. The dynamic treatment effect (DTE) curve typically provides more insight than summary statistics. For instance, we have learned that the inhibitory effect of radiation therapy displays a prominent peak whose timing increases from 10-20 days with radiation dose in the FaDu cell line. In addition, peak inhibition and duration also increases linearly with higher doses of radiation therapy. By contrast, the DTE of Bevacizumab treatment in glioma is qualitatively different: the curve is relatively flat, indicating an approximately constant rate of inhibition for the duration of the study. The relatively unexpected nature of the delayed effect for the combination therapy in glioma suggests that an empirical analysis, as proposed here, should be a first step before developing a mechanistic model.

The shape of the DTE curve indicates the mode of action of a therapy. Curves with positive peaks indicate a cytotoxic therapy. Flat curves suggest a cytostatic mode of action. The shape of the DTE can potentially be exploited to improve the efficacy of a therapy. For instance, if the peak of the DTE curve is too low to cause tumor shrinkage, this might suggest that a higher dose might be required. On the other hand, if the duration of the treatment curve is small, especially if the treatment effect vanishes soon after therapy administration is stopped (such as temozolomide in the combination study), this might suggest that a longer period of therapy is necessary.

In simulation experiments, we have demonstrated that it is possible to obtain a reasonably accurate estimate of the DTE curve with as few as *n* = 10 animals/group, which is reasonable for xenograft studies. Even in situations where data is heavily censored, we have demonstrated it is possible to obtain a consistent estimate using model based imputation via the EM algorithm. More work is required to exhaustively characterize the types of scenarios where consistency is maintained and to improve efficiency of estimates, e.g. using a mixture modelling approach for curves [27]. Although the treatment effect curve derived here is based on the assumption of log-linear growth in untreated tumors, the methodology can be extended to more general modes of growth in untreated tumors, although the estimation and interpretation of effects will become more complex. In keeping with the philosophy that the effect size is more important than its significance in xenograft trials [28], we have omitted statistical inference from this paper. Nevertheless, it is possible to test hypotheses, estimate confidence intervals etc. for either features derived from the DTE curve or the entire curve itself using methods of functional data analysis [16]. For more efficient inference, it may often be possible fit a parametric model whose choice is guided by the shape of the DTE curve.

## MATERIALS AND METHODS

### Estimating the dynamic treatment effect (DTE) curve

Over the entire range from inception to maturity, tumor growth in mice is well modelled by a sigmoid curve [12]. In typical xenograft experiments, however, we only get to observe the ‘middle’ of this sigmoid curve, which represents the fast growth phase. For a wide range of tumors, untreated tumor growth is this phase is well approximated by log-linear growth [3]. In addition, the measurement error of the tumor volume is approximately additive on the log scale [8]. With these assumptions, we can model the log tumor volume as:

(1.2) $$\log V_{i}(t)=\log V_{i}(0)+\lambda_{i}t-R(t)+\varepsilon (t)$$

Where *V*_i_(*t*) is the tumor volume of the *i*-th mouse at time *t*, λ_i_ is the growth rate of the *i*-th tumor, assumed to be a random sample from a Gaussian distribution with mean λ and SD *σ*_ε_, *R*(*t*) is the cumulative inhibition and ε(*t*) is measurement error, assumed to have a Gaussian distribution with mean 0 and SD *σ*_ε_. Our goal is to recover the time effect curve, which is the instantaneous effect of the therapy of tumor growth, given by *r(t)* = *R'(t)*, the rate of change, or derivative of the cumulative inhibition function, given the observed TGD data. Note that while the treatment effect may also have a random subject specific component, it is not possible to separate this from the random effect in the growth process, as they can cancel each other out. For identifiability, we therefore assume the treatment effect to be fixed. We use the penalized least squares criterion Li to estimate the cumulative tumor growth function f:

(1.3) $$L_{i}(f)=\sum_{t=0}^{T}{\left(\log V_{i}(t)-f(t)\right)}^{2}+v{\int_{0}^{T}\left(\frac{\partial^{2}}{\partial u^{2}}f(u)\right)}^{2}du$$

The first term on the right hand side of is the measure of how well the model fits the data (goodness of fit), while the second term measures the ‘roughness’ of the growth function, as measured by the integral of the square of the second derivative. By this metric, a linear function (straight line, as expected under control) will have a second derivative of 0, hence a roughness of 0 (minimum possible value). By contrast, a function that ‘wiggles’ a lot, i.e. twists and turns, will have a high roughness value. The smoothness parameter controls the tradeoff between the goodness of fit to the data and roughness. The function ${\overset{⌢}{f}}_{i}$ which minimizes *L_i_* with over all possible ‘smooth’ growth functions *f* is a smoothing spline which can be calculated from the data [13]. The derivative of this spline estimate, which we denote by ${\overset{⌢}{f}}_{i}\prime$, is also an estimate of *f*' (*t*), the instantaneous rate of tumor growth. In fact the estimated rate of tumor growth approximates the true rate function with an upper error bound dependent on *T*, the number of timepoints at which the tumor is observed and *r* = (*p* – 1)/(2*p* + 1) [14]. The parameter *p* is a measure of smoothness of the tumor growth function *f*, usually quantified by the highest order derivative with a finite mean squared error value, i.e. $E{\left\|f^{\left(p\right)}\right\|}^{2}<\infty$ [15]. Using this approximation property and the form of the tumor growth function given in (1.2), we can write:

(1.4) $${\overset{⌢}{f}}_{i}\prime (t)=\lambda_{i}-r(t)+O(T^{-r})$$

where *r* (t)= *R*' (*t*) is the instantaneous rate of inhibition, also known as the ‘time effect curve’. Averaging this rate estimate across animals *i* = 1,…, n gives us:

(1.5) f¯'(t) = ∑i=1nf^i'(t) = λ¯ − r(t) + n−1O(T−r)

Where $\bar{\lambda }\;=\;n^{-1}\sum \lambda_{i}$ is the average growth rate of untreated tumors, which can typically be estimated from the control arm of the study. We can therefore obtain an estimate of the time effect curve of a treatment as r^(t) = λ¯ − f¯'(t). From (1.5), the accuracy of this estimator depends both on the number of animals receiving this particular treatment as well as the number of timepoints at which they are measured. For comparability, the same value of the smoothness parameter *v* is used for all animals in a study [16]. In results, we demonstrate methodology the accuracy of this estimate via a simulation experiment.

### Additivity of combination studies

There is much recent interest in combining modern targeted therapies to overcome resistance to mono-therapies [17, 18]. It is therefore useful to have a method for assessing which combinations provide added benefit over single therapies. The generalized tumor growth model (1.2) allows a natural definition of additivity of combination therapy if $f(r_{comb}(t))\;=\;f(r_{1}(t))\;+\;f(r_{2}(t))$, where *r*_1_(*u*), *r*_2_(*u*) and *r*_comb_(*u*) are the time effect curves for two monotherapies and the combination therapy respectively. The function *f* is some feature of the curve, such as Peak = max *r*(*t*) or area under the curve (AUC) = ∫r(u)du. This concept of additivity has been previously used in the context of a specific model of tumor growth [19]. Extending this concept, a combination therapy is *synergistic* if $f(r_{comb}(t))\;>\;f(r_{1}(t))\;+\;f(r_{2}(t))$, *sub*-additive if $f(r_{comb}(t))\;<\;f(r_{1}(t))\;+\;f(r_{2}(t))$ and antagonistic if $f(r_{comb}(t))\;<\max\{f(r_{1}(t)),\;f(r_{2}(t))\}$.

### Analysis of censored data

A common problem with fitting curves to TGD data is that chunks of it are often missing, because the tumor volume is smaller than the observable limit or exceeds the upper allowable threshold (Tan et al. 2002). Ignoring the missing data can lead to bias, which could be positive or negative depending on the nature of the missing data [6, 20]. We have therefore developed a method of handling missing data using the expectation- maximization (EM) algorithm, based on a flexible model for the treatment effect curve. The model is based on a Gaussian shape, which requires a peak location (μ), peak height (a) and peak duration (σ) (Figure 3(a)). This model is used in an iterative expectation maximization (EM) algorithm [21] to obtain maximum likelihood estimates of the growth curve, with the iterated steps being:

**E-step**: Obtain a ‘complete version’ of the tumor growth curve by supplementing the observed data with the model fitted values (where data was missing).
**M-step**: Update the model parameters by fitting it to the current version of the ‘complete’ curve using non-linear least squares.

Convergence of the EM algorithm can be slow [22], so we have chosen to stop it after 1000 iterations. Convergence was not very sensitive to choice of starting value (Figure 3(b)), however the embedded non-linear least squares algorithm can fail to converge for certain parameter combinations. If this happens, a modified starting guess is necessary.
